# Revisited Catalytic Hydrogen Evolution Reaction Mechanism of MoS_2_

**DOI:** 10.3390/nano13182522

**Published:** 2023-09-08

**Authors:** Yuhao He, Xiangpeng Chen, Yunchao Lei, Yongqi Liu, Longlu Wang

**Affiliations:** College of Electronic and Optical Engineering & College of Flexible Electronics (Future Technology), Nanjing University of Posts and Telecommunications, Nanjing 210023, China; b20080216@njupt.edu.cn (Y.H.); 1221025306@njupt.edu.cn (X.C.); 1221025307@njupt.edu.cn (Y.L.); 1222025412@njupt.edu.cn (Y.L.)

**Keywords:** catalytic mechanism, edge engineering, defect engineering, phase engineering

## Abstract

MoS_2_ has long been considered a promising catalyst for hydrogen production. At present, there are many strategies to further improve its catalytic performance, such as edge engineering, defect engineering, phase engineering, and so on. However, at present, there is still a great deal of controversy about the mechanism of MoS_2_ catalytic hydrogen production. For example, it is generally believed that the base plane of MoS_2_ is inert; however, it has been reported that the inert base plane can undergo a transient phase transition in the catalytic process to play the catalytic role, which is contrary to the common understanding that the catalytic activity only occurs at the edge. Therefore, it is necessary to further understand the mechanism of MoS_2_ catalytic hydrogen production. In this article, we summarized the latest research progress on the catalytic hydrogen production of MoS_2_, which is of great significance for revisiting the mechanism of MoS_2_ catalytic hydrogen production.

## 1. Introduction

Hydrogen energy is the ultimate environment-friendly energy and the most promising form of energy to replace traditional energy sources such as coal, oil, and natural gas [1,2,3,4,5]. At present, the production of hydrogen mainly relies on the cracking of traditional energy sources, which belongs to false decarbonization [6,7,8]. Hydrogen production by solar photovoltaic power generation is the most promising method of hydrogen production [9,10]. The key to electrocatalytic hydrogen production lies in the development and utilization of an electrocatalyst. Because the precious metal platinum has good catalytic hydrogen production performance, it is the best hydrogen production catalyst at present; however, its high cost and scarce resources seriously hinder its application in catalytic production.

As a non-precious metal catalyst with the most potential to replace precious metal platinum, MoS_2_ has attracted increasing attention [11,12,13,14,15,16]. The key factors that determine the catalytic hydrogen production performance of MoS_2_ mainly relate to two aspects: one is the number of active sites, and the other is the true activity of the active site [17,18,19,20,21,22]. It is generally believed that the base surface of MoS_2_ is chemically inert and does not have the performance of catalytic hydrogen production; moreover, at the same time, the edge of MoS_2_ has high catalytic hydrogen evolution activity [23,24,25,26,27]. More and more people are using various methods to expose the edge of MoS_2_ to improve its catalytic hydrogen production performance [28,29,30,31,32]. The latest research shows that the conversion of sheet MoS_2_ into bands can expose the edge sites and improve the catalytic hydrogen production performance [33,34,35,36,37,38,39,40,41]. It is also possible to directly generate branchlike MoS_2_ by controlling the proportion of precursors during the growth of MoS_2_, thereby increasing the edge site of MoS_2_ [42,43,44,45,46,47,48]. Defect engineering and phase engineering are also strategies to regulate the catalytic hydrogen production performance of MoS_2_ [49,50,51,52,53,54,55,56]. Although the catalytic hydrogen production performance of MoS_2_ can be adjusted through various regulatory strategies, the corresponding catalytic mechanism is still very controversial. It has been reported that the inert base plane can undergo a transient phase transition in the catalytic process to play the catalytic role, which is contrary to the common understanding that the catalytic activity only occurs at the edge [57].

Only a deep and correct understanding of the catalytic mechanism of MoS_2_ can further promote the design of high-performance MoS_2_ structures, so as to promote the improvement of its catalytic performance. With the help of first principles theoretical calculation and in situ characterization, the catalytic process can be understood from the molecular and atomic levels and the mechanism of catalytic hydrogen production can be revealed. In this review, we introduce the latest research progress of strategies to improve the performance of MoS_2_ catalytic hydrogen production and give prospects for the development and direction of this research field.

## 2. Edge

It is well known that the edge site of MoS_2_ has high catalytic HER activity, and a lot of research has focused on how to expose the edge of MoS_2_. Recent studies have shown that the MoS_2_ can be designed with a rich edge structure such as a paired edge nanoribbon, which can further enhance the catalytic HER activity of MoS_2_.

### 2.1. Nanoribbon

According to research on edge-dominated electrochemical reaction kinetics in ultra-narrow MoS_2_ nanoribbons, ideal energetics for HER could be obtained. Large arrays of MoS_2_ nanoribbons were acquired using a templated subtractive patterning process (TSPP), which significantly enhanced the turn-over frequency, exchanged the current density, and lowered the Tafel slope because of improved charge transfer efficiency.

**Figure 1:**
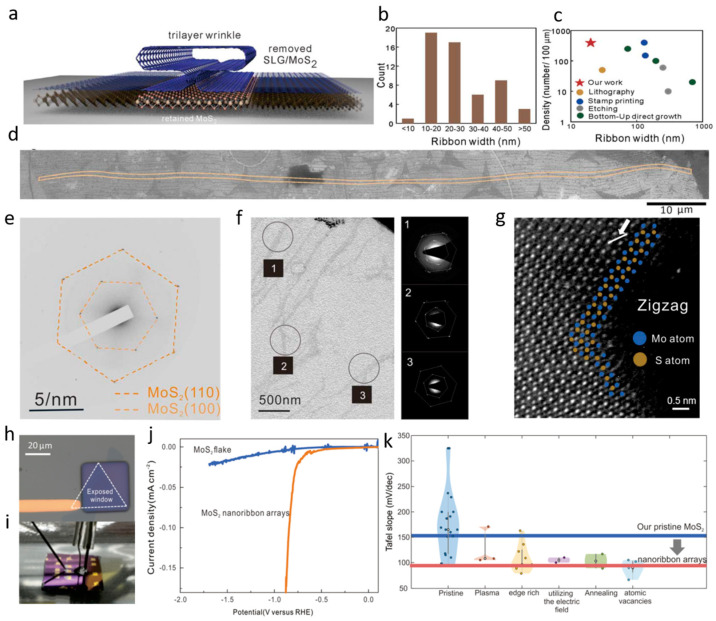
(**a**) Schematic representation of the wrinkle-templated nanoribbon formation. (**b**) A histogram that depicts the width distribution of nanoribbons was determined through an analysis of transmission electron images. (**c**) A comparison between the attainable width and array density of nanoribbons in this study and previous results. (**d**) Composite SEM image with a nanoribbon exceeding 100 μm. (**e**) A selected area diffraction pattern that indicates the symmetry of MoS_2_. (**f**) A low-resolution TEM image with SAED patterns, demonstrating that three distinct areas exhibit long-range atomic alignment. (**g**) An atomic resolution TEM image reveals the orientation of the lattice structure. (**h**) An optical micrograph displays a monolayer of 2H-MoS_2_, showing an exposed reaction window and a gold contact. (**i**) A photograph illustrating the capillary microcell employed for HER measurements. (**j**) Comparison between the performance of a MoS_2_ flake and a nanoribbon array using polarization curves. (**k**) A comparative analysis between literature values of pristine and modified 2H-MoS_2_ and the results obtained in this study. Adapted with permission from [53], Copyright 2023 Royal Society of Chemistry.

Utilizing the naturally occurring bilayer and multilayer regions in graphene, and taking advantage of the bottom-up approach of graphene, the pattern is transferred from the graphene mask to the surface of the MoS_2_ material through a pattern transfer process, thus forming an aligned MoS_2_ nanoribbon array with a controlled direction, as shown in Figure 1a. Since the formation of nanoribbons is random to a certain extent, the width distribution is also affected in Figure 1b. With a length-to-width ratio of more than 7000 and a high density (Figure 1c), the strips are more efficient than other strategies for patterning MoS_2_ nanoribbons. The observation of a single nanoribbon over a long distance in Figure 1d shows that the fractures are solved and the structural stability of the nanoribbon is ensured. Using the electron diffraction technique, the crystal properties of MoS_2_ nanoribbons can be determined and characterized. The six-fold symmetry diffraction pattern in Figure 1e was observed using the SAED model. Through an analysis of the multiple SAED patterns presented in Figure 1f, the orientation of the nanoribbon was determined not to affect the crystal structure. The atomic arrangement in the TEM in Figure 1g shows an orderly structure with no obvious defects. The difference in brightness may be due to the atomic number of the atoms; the darker atoms are Mo and the brighter ones are of S. High basal plane quality, which makes their nanoribbon array an ideal model system for studying the source of HER enhancement. A three-electrode localized electrochemical microcell technique was employed to conduct electrochemical research. Consisting of an exposed reaction window and gold contact, as shown in the schematic of the microelectrode structure in Figure 1h,i, it may be used to place droplets for electrochemical studies. As shown in Figure 1j, the HER exchange current of the MoS_2_ nanoribbon arrays is significantly larger than that of pristine flakes, and the overpotential shows a decrease of 41%, which reveals an improved HER thermodynamic performance. Because the device is not deposited on a conductive surface and the carrier passes laterally through the nanoribbons from the electrode, introducing an uncompensated resistance, HER kinetics is quantified with the fitted Tafel curve. The Tafel slope of the pristine flake is consistent with previous results. As a comparison, the nanoribbon arrays’ Tafel slope in Figure 1k greatly decreased, showing the impact of edges on the improved HER kinetics of MoS_2_. It can also be pointed out that the observed Tafel slope is in good agreement with prior findings on edge-enriched, 2D materials, and it exhibits a clear distinction from alternative functionalization approaches.

### 2.2. Fractal MoS_2_

Since the catalytic active site of 2H-MoS_2_ is mainly at its edge, controlling the morphology and structure of MoS_2_ to expose more edges can further improve the hydrogen evolution reaction (HER) of MoS_2_. Then, if MoS_2_ is grown in a multi-branched and multi-edge morphology structure in the chemical vapor deposition (CVD) growth process, the HER performance of MoS_2_ can be improved. Therefore, Yu G. et al. synthesized MoS_2_ with different morphologies by adjusting the proportion of precursor in the process of MoS_2_ generation by CVD.

**Figure 2:**
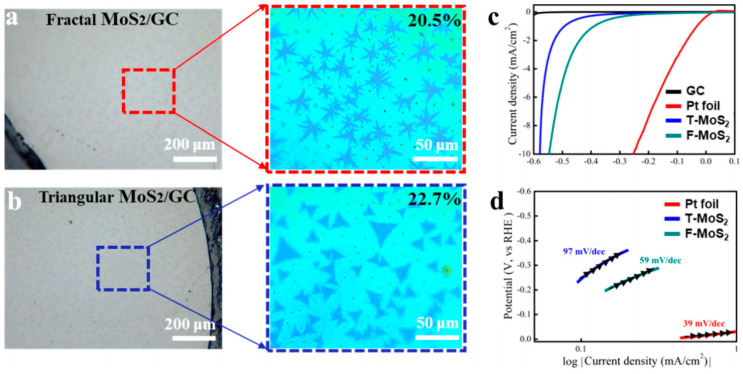
(**a**,**b**) Optic microscopy (OM) images of different kinds of MoS_2_ transferred to the glassy carbon (GC) electrode, (**c**) polarization curves of the two MoS_2_ samples, and (**d**) the Tafel slope diagram. Adapted with permission from [54], Copyright 2022 American Chemical Society.

As shown in Figure 2a,b, fractal MoS_2_ and triangular MoS_2_ were obtained by controlling the proportions of MoO_3_ and S, respectively, and the coverage rate of fractal MoS_2_ and triangular MoS_2_ was determined to be 20.5% and 22.7% using image analysis software. When MoO_3_ is sufficient, a triangular MoS_2_ can be generated, while, at a low dose of MoO_3_, a fractal MoS_2_ will be generated. After the formation of MoS_2_ with different morphologies, their catalytic properties were further evaluated. Figure 2c shows the polarization curves of the two MoS_2_ samples, the GC electrode and Pt foil. Compared with triangular MoS_2_, fractal MoS_2_ has a smaller initial hydrogen evolution overpotential; moreover, as shown in Figure 2d, the Tafel slope of the fractal MoS_2_ is lower than that of the triangular MoS_2_. It is therefore confirmed that the fractal MoS_2_ has more active edges and better catalytic activity.

## 3. Sulfur Vacancies

Vacancies are considered to be the limiting doping states that promote atomic rearrangements and modulate the electronic structure over a wide range. Many methods have been successfully implemented to introduce vacancies in 2D TMDs, such as hydrogen plasma exposure, H_2_ annealing, Ar^2+^ beam irradiation, and helium ion beam irradiation, showing great potential for catalytic reactions. However, all of the above methods require additional intervention from external stimuli; therefore, it is difficult to generate controllable vacancies directly by growth.

Defect engineering is an effective strategy to accelerate the catalytic hydrogen production performance of MoS_2_. However, introducing defects such as sulfur vacancies on the MoS_2_ basal plane is still a major challenge. Currently, sulfur vacancies are mainly introduced into MoS_2_ by using post-treatment methods such as plasma treatment, ultrasonic, ball milling, and other methods. However, if sulfur vacancies can be introduced directly during the preparation of MoS_2_, it would be an excellent strategy to prepare sulfur vacancy defects.

**Figure 3:**
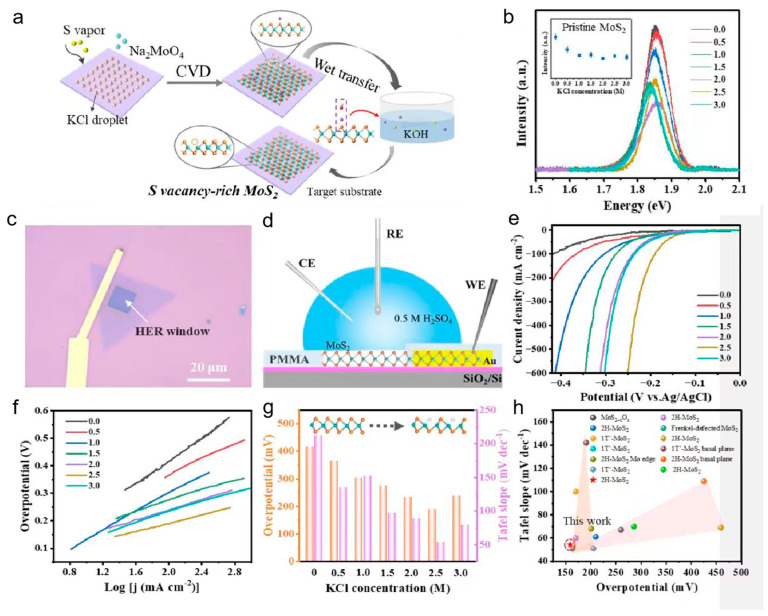
(**a**) Schematic illustration of the CVD growth of vacancy-rich MoS_2_. (**b**) Spectra of MoS_2_ basal plane under various KCl concentrations from 0.0 to 3.0 M. The inset shows the statistical results of PL intensity under each concentration (C_KCl_). (**c**) Optical image and (**d**) schematic of the as-fabricated MoS_2_ microdevice. WE: work electrode, RE: reference electrode, CE: counter electrode. (**e**) LSV curves and corresponding (**f**) Tafel plots of the MoS_2_-KCl microdevices. (**g**) Comparison of overpotentials (red) and Tafel slopes (orange) under different KCl concentrations. (**h**) A comparison between the Tafel slope and overpotential of this work and the reported MoS_2_-based catalysts. Adapted with permission from [55], Copyright 2023 Wiley-VCH GmbH.

As shown in Figure 3a, Man et al. proposed that sulfur vacancies can be introduced into the MoS_2_ basal plane by controlling the reaction conditions during the MoS_2_ growth through a salt-assisted CVD method [55]. The density of sulfur vacancies could be controllable by controlling the added amount of KCl during the CVD growth process, and some kind of change has occurred during the process. Figure 3b shows the luminescence spectra of the obtained MoS_2_ with different densities of sulfur vacancies, and it was found that the luminescence intensities of the obtained samples were different when the added amount of KCl was different, which indicates that the density of sulfur vacancies is positively correlated with the added amount of KCl. It is noted that the energy band holds steady when the added KCl reaches a certain amount. The density of sulfur vacancies could be successfully controlled using this salt-assisted CVD method. In order to explore the relationship between the density of sulfur vacancies and the catalytic hydrogen production performance of MoS_2_, a micro-nano HER test platform was built (shown in Figure 3c,d) to precisely evaluate the catalytic performance of monolithic MoS_2_ with sulfur vacancies. It was found that the samples with abundant sulfur vacancies had the best catalytic hydrogen production performance and the lowest Tafel slope (Figure 3e,f). The overpotential was negatively correlated with the concentration of added KCl (Figure 1g), which confirmed that the sulfur vacancies of MoS_2_ could be active sites for catalytic hydrogen production. The catalytic hydrogen production performance becomes much better when the density of sulfur vacancies is higher. Figure 1h shows the relationship between the overpotential and Tafel slope of all the samples along with sulfur vacancy as reported in other literature. It could be seen that the sample obtained by this work has the best catalytic performance, which indicates that the salt-assisted CVD method is an excellent strategy for creating the sulfur vacancies that serve as HER catalytic active sites.

**Figure 4:**
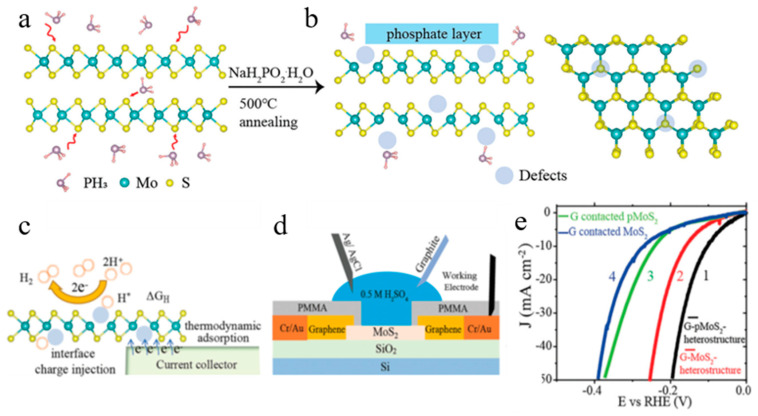
(**a**) Schematic of PH_3_ molecule extracting sulfur atoms from the MoS_2_ layers. (**b**) The defects’ formation in the basal planes, point/line defects, and edges, resulting in H_3_PO_4_ layers on top of MoS_2_ catalysts. (**c**) Schematic comparison of thermodynamic hydrogen adsorption and interface charge injection that determined the performance of hydrogen evolution reaction. (**d**) Schematic cross-section view of monolayer graphene and PH_3_-treated monolayer MoS_2_ electrochemical device. (**e**) Normalized polarization curves measured from different graphene and MoS_2_ devices. Adapted with permission from [56], Copyright 2023 Wiley-VCH GmbH.

The method of thermochemical annealing sodium hypophosphite to produce MoS_2_-active defects is proposed; meanwhile, it can spontaneously produce PH_3_ to regulate the MoS_2_ lattice. By controlling the reaction conditions, active defects are formed at the basal plane and edges, thereby exposing more metal active sites and improving the Hydrogen Evolution Reaction (HER) performance of MoS_2_. The development of efficient and low-cost MoS_2_ catalysts for practical applications is important. Sodium hypophosphate is set at around 200 °C to produce PH_3_ gas, and MoS_2_ is annealed using PH_3_ gas at 500 °C as shown in Figure 4a. PH_3_ reacts with MoS_2_ to produce defects that replace the S atom in the MoS_2_ lattice through defects, resulting in P doping (Figure 4b). Due to the active chemical properties of doped P, the active P element can be oxidized to a phosphate layer coating on the surface of MoS_2_ and form phosphate without phosphate compounds in the MoS_2_ crystal. The phosphate could be eventually removed from the crystal lattice of the MoS_2_ crystal if it is dissolved in water or acid solution, thus creating defects again. Active defects may provide additional adsorption sites or change the local environment of the atom. When the proton is adsorbed to active defects from a relatively stable state, the ΔGH* (Gibbs free energy) of the system will change to regulate the thermodynamic adsorption/desorption of the proton. Energy level inhomogeneity can also be introduced to regulate the interface energy level and facilitate electron transport, which ultimately optimizes HER activity (Figure 4c). To further explore the more important factors affecting HER activity, a microelectrochemical reactor was used to distinguish the influence of interfacial charge injection and thermodynamic adsorption. As shown in the schematic, this is the cross-section of monolayer graphene and a PH3-treated monolayer MoS_2_ electrochemical device (Figure 4d). The performance of a single MoS_2_, rather than the whole catalyst, can be directly investigated in a microelectrochemical reactor, and the enhanced HER activity due to specific factors can be demonstrated. Graphene and unannealed/annealed MoS_2_ monolayer nanosheets were prepared as contact electrodes and target catalysts, respectively. Comparing the relative overpotential of the four devices, it was found that the overpotential of the graphene-pMoS_2_ heterostructure device was −100 mV at 10 mA·cm^−2^, which was much smaller than that of the other three devices, and the Tafel slope gradually decreased from curve 4 to 1 (Figure 4e). The difference of curves in overpotential caused by charge transfer for 3 to 4 is −32 mV, while 1 to 2 is −60 mV. The thermodynamic ΔG_H_* is −140 mV for 1 to 3 and −112 mV for 2 to 4. It can be concluded from the data that charge transfer plays a less important role than thermodynamic ΔG_H_* due to drastic changes in properties.

## 4. Doping

Doping engineering has become an effective strategy to improve MoS_2_ base activity [57]. The most common doping engineering practice is to improve its electronic structure through heteroatom doping, thereby enhancing its intrinsic catalytic activity. This is mainly because the electronic structure of the base atoms is modulated using defect engineering to improve its surface conductivity [58]. In addition, heteroatom doping has important effects on chemical bond formation, adsorption/desorption processes, and the Gibbs free energy of the reaction [59,60,61,62]. Therefore, the electrocatalytic activity of MoS_2_-based catalysts can be effectively improved according to the electronegativity difference and the type and number of heteroatoms.

1T-phase MoS_2_ (1T-MoS_2_) has been widely concerned in hydrogen evolution reaction (HER) because it exhibits better charge transport characteristics and can expose more active sites. Although 1T-MoS_2_ is a good HER material under acidic conditions, it produces a higher overpotential under alkaline conditions. At the same time, the conditions of an alkaline solution are more suitable for HER.

**Figure 5:**
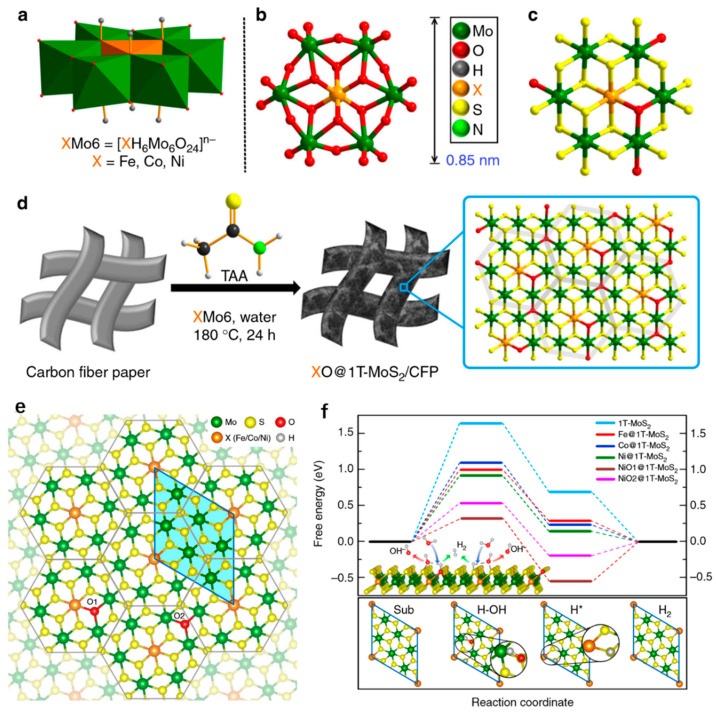
(**a**) Polyhedral characterization of XMo6 precursors. (**b**) Spherical and rod-like characterization of XMo6 precursors. (**c**) Mode structure of XO@1T-MoS_2_. (**d**) Diagram of the preparation of atomic scale transition metal and oxygen co-doped 1T-MoS_2_ nanosheets on carbon fiber paper (CFP) using incomplete vulcanization of XMo6. (**e**) Single-layer structure of X@1T-MoS_2_ formed by O co-doped XMo_6_ hexagonal primitive. (**f**) Surface free energy diagram of different catalysts in alkaline solution. Adapted with permission from [63], Copyright 2019 Nature Publishing Group.

The design and modification of the catalytic site at the atomic level can deepen the understanding of the active site of the catalyst, which is essential to enhance the activity of the catalyst. In this regard, Jing Gu et al. used Anderson-type polyoxometalates as a precursor to doping the metal active site onto 1T-MoS_2_ at the atomic level to improve the HER activity of 1T-MoS_2_. As shown in Figure 5a,b, the precursor Anderson-type POM nanoclusters, [XH_6_Mo_6_O_24_]^n−^ (denoted as XMo_6_, where X represents the doped metal atoms such as Fe, Co, Ni, etc.) is characterized by different shapes. Figure 1c shows the resultant XO@1T-MoS_2_ structure. Figure 5d shows the synthesis process using XMo_6_ to construct XO@1T-MoS_2_ on highly conductive carbon fiber paper (CFP) by vulcanizing the hydrothermal reaction. It is generally considered that ΔG (H_2_O) ΔG (H*) is important for understanding HER catalytic activity in the alkaline medium HER process. Using the density functional theory (DFT) calculation, the structure of XO@1T-MoS_2_ and the free energy diagram of the catalyst surface during the reaction were constructed. As shown in Figure 5e, the structure of XO@1T-MoS_2_ consists of a hexagonal XMo_6_S_14_ unit with six Mo atoms surrounding the X atom. As shown in Figure 5f, the calculated Gibbs free energy shows that the ΔG (H*) of 1T-MoS_2_ doped with transition metal is significantly reduced. The ΔG (H*) of Ni@1T-MoS_2_ is close to 0, indicating that it can promote the desorption of H*. At the same time, the addition of transition metals also significantly reduces ΔG (H_2_O) and promotes the dissociation of H_2_O to H*. Then, the synergistic effect of oxygen and Ni was studied by controlling the amount of oxygen incorporation. It was found that ΔG (H_2_O) and ΔG (H*) were further reduced with the addition of oxygen, while excess oxygen would increase them. In summary, the HER properties of the catalyst can be promoted by incorporating transition metal and appropriate oxygen into 1T-MoS_2_.

## 5. Phase

It is generally believed that the base plane of MoS_2_ is inert; however, it has been reported that the inert base plane can undergo a transient phase transition in the catalytic process to play the catalytic role, which is contrary to the common understanding that the catalytic activity is only at the edge. The HER catalytic mechanism of 1T-MoS_2_ remains elusive and controversial. Therefore, it is necessary to further understand the mechanism of MoS_2_ catalytic hydrogen production.

### 5.1. An Irreversible Phase Transition during Photocatalytic Hydrogen Evolution

It is widely believed the active sites of 2H-MoS_2_ for catalytic hydrogen production are located at the edges, while its basal plane is inert. Moreover, it has been reported that the conversion of the 2H phase into the 1T phase by phase transformation is an ideal strategy to enhance the catalytic HER performance of MoS_2_. However, the HER catalytic mechanism of 1T-MoS_2_ remains elusive and controversial. It is difficult to explain the nature of the better catalytic performance, which is originally from the improved electrical conductivity, the increased intrinsic activity of the active site, or the number of active sites.

**Figure 6:**
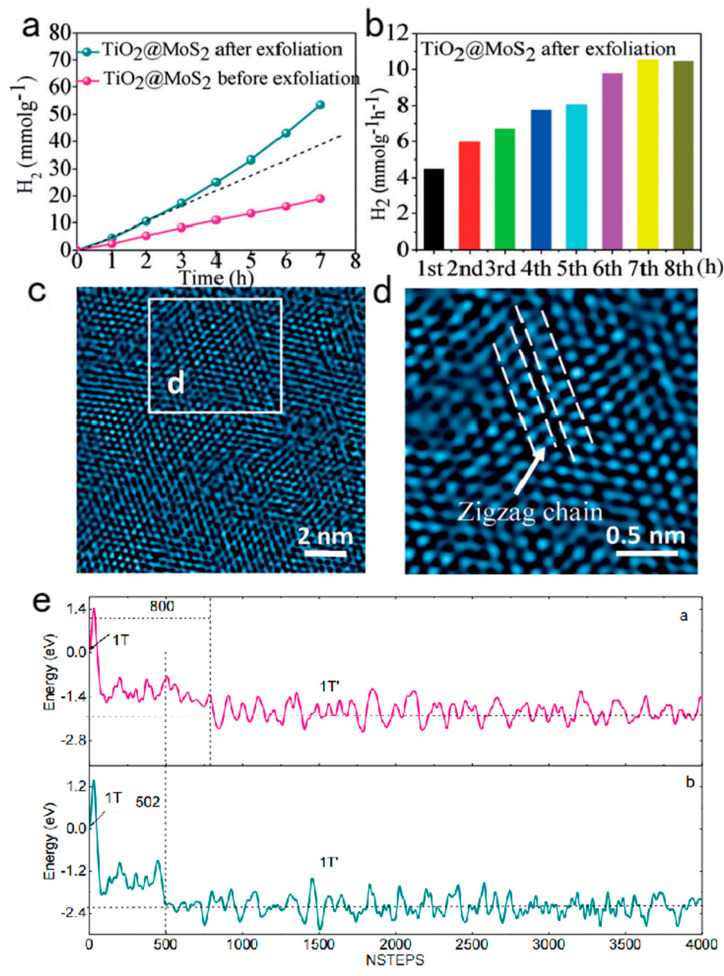
(**a**) H_2_ accumulation and (**b**) H_2_ production rate over exfoliated TiO_2_@MoS_2_ at each hour of the HER. All experiments were carried out in 80 mL of 15% (*v*/*v*) TEOA aqueous solution under visible light irradiation (λ > 420 nm). Catalysts: 20 mg and EY: 20 mg. (**c**) HRTEM image of the MoS_2_ in the exfoliated TiO_2_@MoS_2_ after 7 h HER. (**d**) Enlarged view of the square in c (the arrow points to the zigzag chain configuration of 1T’). (**e**) Evolution of total electronic energy using AIMD simulations in the MoS_2_ phase transformation at 298 K. (a—1T phase MoS_2_ transform into 1T’ phase spontaneously; b—1T phase MoS_2_ with 1/16 H coverage transform into 1T’ phase). Adapted with permission from [13], Copyright 2017.

In order to explore this problem, the Wang group made ultra-thin MoS_2_ nanosheets that were vertically grown on TiO_2_ nanofibers, and this vertical growth could introduce the strain. The 1T-MoS_2_ with sulfur vacancies and strain could be obtained by further lithium intercalation. As shown in Figure 6a, using this sample as the catalyst for HER, it was found that its catalytic performance gradually increased during the process of catalytic hydrogen production. It was also found that the catalytic hydrogen production per hour increased gradually with time in Figure 6b. This self-optimization of the catalytic performance is most likely due to the structural transformation of the catalyst during the catalytic HER process. In order to investigate this transformation, the catalyst after the catalytic reaction was structurally traced. The HRTEM in Figure 6c,d shows that the 1T phase has transformed into the 1T’ phase, with the super-lattice structure from the Mo atom clustering into Zigzag chains. This suggests that the 1T’ phase is the true active phase for catalytic HER. A molecular dynamics simulation was performed to research the transition from the 1T phase to the 1T’ phase (Figure 6e). It was found that the 1T phase with surface-adsorbed hydrogen was more easily converted to the 1T’ phase, which means that the surface-adsorbed hydrogen could promote the transformation of the 1T phase to the 1T’ phase. This is sufficient to show that, in the photocatalytic HER process, the 1T phase with adsorbed hydrogen atoms on the surface could transform into the 1T’ phase with high activity, which leads to the phenomenon of the self-optimization of the catalytic performance. This work therefore reveals the catalytic mechanism of 1T-phase MoS_2_.

### 5.2. Transient Phase Transition during the Hydrogen Evolution Reaction

**Figure 7:**
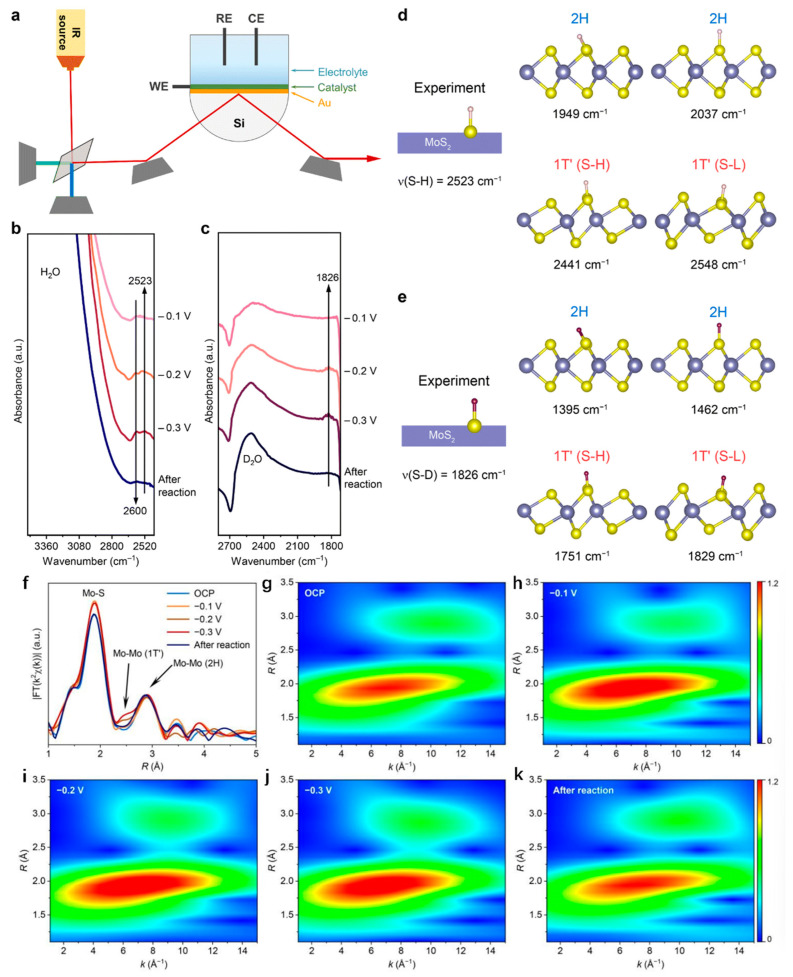
(**a**) Schematic diagram of a three-electrode ATR-SEIRAS cell measured in situ. (**b**) ATR-SEIRAS spectra of −0.1 V, −0.2 V, −0.3 V, and after the reaction measured in 0.5 M H_2_SO_4_ electrolyte and (**c**) 0.5 M D_2_SO_4_ electrolytes. (**d**) Comparison of ν(S-H) experimental data and calculated data of 2H-MoS_2_ and 1T’-MoS_2_. (**e**) Comparison of experimental and calculated data of ν(S-D) of 2H-MoS_2_ and 1T’-MoS_2_ after electrolyte replacement. The S atom is divided into S-H and S-L according to its position on 1T’-MoS_2_. (**f**) In situ EXAFS spectra of 2H-MoS_2_ with respect to reversible hydrogen electrodes (RHEs) at OCP, −0.1 V, −0.2 V, and −0.3 V and after reaction. (**g**–**k**) Wavelet transform (WT) analysis of 2H-MoS_2_ at (**g**) OCP, (**h**) at −0.1 V, (**i**) at −0.2 V, (**j**) at −0.3 V, and (**k**) after the reaction. Adapted with permission from [64], Copyright 2023 Royal Society of Chemistry.

2H-MoS_2_ is one of the most promising noble metal-free electrocatalysts in the hydrogen evolution reaction (HER). With regard to its HER mechanism, the widely accepted view, so far, is that its marginal sites have high HER activity, while its basal plane is inert during the HER process. However, Zhai et al. found that this conclusion was incorrect and verified it using ATR-SEIRAS and XAFS. The three-electrode ATR-SEIRAS cell used for the in situ measurement is shown in Figure 7a. As shown in Figure 7b, 0.5M H_2_SO_4_ was added to the three-electrode ATR-SEIRAS cell as the electrolyte, and the ATR-SEIRAS spectra of MoS_2_ at −0.1 V, −0.2 V, −0.3 V and after reaction were measured. It can be found that a peak of 2523 cm^−1^ occurs at −0.2 V and −0.3 V, and this peak is not from the edge site; rather, a continuous peak of 2600 cm^−1^ is from the edge site. The formation of the S–H bond was observed at −0.2 V, indicating that 2523 cm^−1^ in the experiment is the stretching vibration of the S–H bond (v(S-H)). Then, as shown in Figure 7c, the stretching vibration of the S–H bond is calculated and compared with the experimental value. There are two adsorption modes of the S–H bond in 2H-MoS_2_, namely, vertical adsorption and inclined adsorption. However, the calculated values of the v(S-H) of the two models differ greatly from the experimental results. Similarly, the S–H bond in 1T′-MoS_2_ also has two adsorption modes, which are, respectively, in the higher position and the lower position and are denoted as S-H and S-L. It is found that v(S-H) on S-L has a good agreement with the experimental value. In addition, as shown in Figure 1d,e, when the electrolyte H_2_SO_4_ in the experiment was replaced by D_2_SO_4_, that is, when the proton source in the HER process was replaced by D, the conclusion remained unchanged, and v(S-H) on S-L also had a good agreement with the experimental value. The experimental results show that there is a phase transition from 2H to 1T′ during the reaction. As shown in Figure 7f–k, in order to further verify that the 2H to 1T′ phase transition is not permanent but transient, the EXAFS diagram during the reaction process and the wavelet transform analysis of 2H-MoS_2_ were measured. As shown in Figure 7f, under open-circuit potential (OCP), the length of the Mo–S bond and Mo–Mo bond is consistent with those of 2H-MoS_2_, while the length of the Mo–Mo bond is consistent with 1T′-MoS_2_ at −0.2 V and −0.3 V. It is found that the characteristics of 1T′-MoS_2_ disappear after the end of the reaction, indicating that this phase transition is transient. As shown in Figure 7g–k, the WT data further indicate that this phase transition is transient, and no 1T’-MoS_2_ feature appears at OCP and −0.1 V, and the 1T’-MoS_2_ feature appears at −0.2 V; moreover, this feature is more obvious at −0.3 V, and, when the reaction ends, the feature of 1T’-MoS_2_ disappears. In conclusion, Zhai et al. verified that part of the base phase of 2H-MoS_2_ would change into 1T’-MoS_2_ during the HER process, showing high activity, and that 1T’-MoS_2_ would change into 2H-MoS_2_ after the reaction.

## 6. Conclusions and Outlook

A number of strategies have been developed to improve the catalytic production performance of MoS_2_, and the mechanism of MoS_2_ catalytic hydrogen production has also been proposed. It is necessary to summarize and reconsider the latest mechanism of catalytic hydrogen production. In this review, we summarized the latest strategies to improve the catalytic hydrogen production of MoS_2_ and the mechanism of catalytic performance improvement. We believe that, in this research field, it is necessary to further promote the improvement of MoS_2_ catalytic hydrogen production performance from the following aspects. The catalyst with an ideal atomic structure should be prepared in view of the controversy over the mechanism of MoS_2_ catalytic hydrogen production, and the catalyst should be used as a model to explore the mechanism of catalytic hydrogen production, combined with first-principles calculation and in situ characterization methods. The catalytic hydrogen production performance of MoS_2_ should be standardized by constructing a micro-nano structure device, and the catalytic hydrogen production performance should be attributed to the catalytic active site with a specific atomic structure.

## Data Availability

Not applicable.
